# Assessing Antibiotic Residues in Poultry Eggs from Backyard Production Systems in Chile, First Approach to a Non-Addressed Issue in Farm Animals

**DOI:** 10.3390/ani10061056

**Published:** 2020-06-19

**Authors:** Javiera Cornejo, Ekaterina Pokrant, Francisco Figueroa, Ricardo Riquelme, Pablo Galdames, Francisca Di Pillo, Pedro Jimenez-Bluhm, Christopher Hamilton-West

**Affiliations:** 1 Food Safety Laboratory, Department of Preventive Veterinary Medicine, Faculty of Veterinary Science, Universidad de Chile, Santiago 8820808, Chile; jacornej@uchile.cl (J.C.); katiavalerievna@ug.uchile.cl (E.P.); francisco.figueroa@ug.uchile.cl (F.F.); ricardo.riquelme@uchile.cl (R.R.); 2 Epidemiology Unit, Department of Preventive Veterinary Medicine, Faculty of Veterinary Science, Universidad de Chile, Santiago 8820808, Chile; pgaldames@veterinaria.uchile.cl (P.G.); pedrojimenezb@gmail.com (P.J.-B.); 3 Nucleo de Investigaciones Aplicadas en Ciencias Veterinarias y Agronómicas, Facultad de Medicina Veterinaria y Agronomía, Universidad de Las Américas, Sede Providencia, Manuel Montt 948, Santiago 7500972, Chile; fdipillo@udla.cl

**Keywords:** food safety, antimicrobials, backyard poultry, poultry eggs, Chile

## Abstract

**Simple Summary:**

Eggs are a readily available and important food source for low income families that raise chicken in their households. It is therefore important to monitor if these products are safe for human consumption and contain no antibiotic residues that could lead to allergic reactions, intoxication, or antimicrobial resistance. However, little is known about the antimicrobial content of eggs consumed in rural households in Chile. Consequently, the aim of this study was to collect chicken eggs from rural households in the central region of Chile and screen them for antimicrobial activity. The results indicate that most collected eggs (73% of the 83 surveyed households), exhibited antimicrobial activity for at least one of the four tested antimicrobials. These results indicate that household members who consume these eggs are in danger of developing antibiotic-related illnesses and could contribute to antimicrobial resistance. Therefore, further studies are required to identify the exact compounds used to treat the chickens and to establish preventive measures to eradicate antimicrobial presence in their food supply.

**Abstract:**

Eggs are the main product generated from backyard poultry production systems (BPS) because they can quickly be consumed and sold to meet essential family needs. Nevertheless, antimicrobial residues can accumulate in this product. The objective of this study was to evaluate the presence of antimicrobial residues in eggs produced by poultry kept in BPS in central Chile. To assess this, eggs were obtained from 83 BPS and analysed to evaluate the presence of antibiotic residues (families: tetracyclines, beta-lactams, aminoglycosides and macrolides), using a Four-Plate Test screening method for the detection, based on a bacterial growth inhibition method. Results show a lack of biosecurity procedures at BPS level, making these systems susceptible to the dissemination of antimicrobial residues. These include intensive animal production units in the proximity, and the presence of shared watercourses with other farms. Furthermore, 66% of the surveyed owners are indicated as giving pharmacological treatments to their chickens. Eggs from 61 BPS were positive for at least one antimicrobial. Fifty-three BPS were positive for more than one antimicrobial, and one BPS was positive for all four antimicrobials tested. Consequently, there is a risk that poultry eggs produced in BPS in central Chile carry residues of different families of antimicrobials.

## 1. Introduction

Small-scale poultry production is a very common practice in rural households around the world, and several studies have evidenced that these production systems have played and continue to play an important domestic socio-economic role in many poor rural households [1,2]. Backyard poultry production systems (BPS) are the most popular systems within small-scale poultry production, where animals are usually kept without proper disease prevention or control strategies and inadequate management practices [3]. The popularity of these production systems is based on the fact that these animals require minimum investment and contribute to the food security of the households by delivering high-quality animal-source food (eggs and meat). In addition, the products generated in the form of live chicks, meat and eggs can be quickly and easily sold to meet essential family needs [2,4]. However, the precarious conditions described in BPS translate into challenges for households. The lack of biosecurity measures described in these systems has attracted the attention of researchers due to the apparent higher risk of presenting infectious diseases such as Highly Pathogenic Avian Influenza (HPAI), Newcastle Disease (ND), Salmonellosis, Gumboro disease, among others [3]. Although there are studies focused on the presence of pathogens in live animals [5], little is known about the food safety of the products generated by these animals in these systems.

Recent studies performed in Chile have characterized animal health management and the contribution of BPS to food access [2], and the presence of avian influenza virus, *Salmonella* spp. and bacteriophages with the ability to lyse *Salmonella* serovars have been identified in these production systems [6,7,8,9,10,11].

Regarding the use of antimicrobials in small-scale farm production in Chile, it was recently reported that the adoption level of good practice protocols of the use of veterinary drugs among peasant family farmers in Chile was mid- or low. These practices include the use of officially authorized products, the acquisition of veterinary drugs with prescription, the drugs’ application in the recommended productive stage, and knowing and respecting withdrawal and re-entry periods [10]. In addition, a previous study identified that in most BPS (72%), no treatment of sick birds or preventive procedures against diseases were applied. However, 8% of the studied BPS reported treating birds using medicinal plants or drugs registered for other animal species than poultry (including drugs for human use) [4]. Meanwhile, another study analysed the presence of antimicrobials in food products sourced from small-scale productions and found trace concentrations of antimicrobials in foodstuffs produced by peasant family farmers in Chile [12]. In Chile, the Official Veterinary Service regulates the registration and marketing of antibiotics for animal use, so access to them is not so easy. However, the authors’ experience in the field has identified that farmers give their birds antibiotics for human use, or they use antibiotics for animal use that were left after the treatment of other animal species they have at home [2].

In relation to this, antimicrobial residues can accumulate in egg white or yolk when administered therapeutically by the use of medicated feed or when the diet of hens is accidentally contaminated [13]. The appearance of antimicrobials in yolk and albumen highly depends on the pharmacokinetic properties of the drug used, so the distribution and deposition pattern of residues will vary for each antimicrobial agent [14,15]. Several studies have evidenced the presence of different antimicrobials in small-scale farm produced eggs in developing countries [16,17,18,19,20]. However, there are no studies assessing the presence of antimicrobials in small-scale egg production in Chile.

Considering that the egg is the product most consumed and marketed from BPS [2,4], the study of the presence of antimicrobials residues in eggs provided from small producers is needed in order to assess their impact in public health. In this way, the objective of the present study was to evaluate the presence of antimicrobial residues in eggs produced by domestic chickens kept in BPS in central Chile.

## 2. Material and Methods

### 2.1. Samples Collection and Farm Data Collection

For this study, eggs were obtained from domestic chickens kept in 83 BPS in central Chile (regions of Valparaiso and Libertador General Bernardo O’Higgins). According to the last census, there are more than 20,000 BPS and more than 540,000 backyard poultry in that area. The eggs were collected during surveillance activities related to influenza virus, based on the willingness of the producer to share the eggs (convenience sampling) [21]. Once the eggs were obtained, they were transported in cardboard boxes in a thermal box to the Food Safety Laboratory (LIA) of the Faculty of Veterinary Medicine (University of Chile), where the eggs were transferred individually to 50 mL falcon tubes and stored at 4 ℃ until further analysis.

Additionally, a survey was applied through a personal interview with the aim of obtaining data regarding biosecurity and animal management. The data generated in this survey helped to characterize the productive systems. Furthermore, these data were analysed to identify possible risk factors for the presence of antimicrobial residues in eggs. For this latter objective, a univariate logistic regression was performed using Infostat statistical software.

### 2.2. Chemicals, Reagents and Cultures Media

For the analysis of lyophilized cultures of *Bacillus cereus* ATCC 11778, *Bacillus subtilis* ATCC6633 and *Kocuria rhizophila* ATCC9341 were purchased from Microbiologics^®^ (MN, USA). BD Difco™ Dehydrated Culture Media: Antibiotic Medium 1, Antibiotic Medium 5 and Antibiotic Medium 8; were used in this experiment since they are useful in determining antibiotic potency by microbiological analysis techniques. The BD Difco™ Dehydrated Culture Media were purchased from Becton, Dickinson and Company (Franklin Lakes, NJ, USA). Brain Heart Broth (BHB) was purchased from Biokar Diagnostic (Solabia Group^©^, Pantin, France). Standards of oxytetracycline, erythromycin, ampicillin and amikacin, used for fortified control, were purchased from Sigma Aldrich Inc. (Merck KGaA, Darmstadt, Germany).

Citric-acetone buffer used in the analysis was prepared with acetone, solution A and distilled water (35, 35 and 30 mL, respectively). Solution A was prepared with a solution of citric acid monohydrated 0.2 M and a solution of potassium hydroxide 0.5 M at a ratio of (1:1). Citric acid, potassium hydroxide and acetone were purchased from Merck KGaA (Darmstadt, Germany).

### 2.3. Microbiological Assay

Egg samples were analysed to evaluate the presence of antibiotic residues, using a Four-Plate Test screening method for the detection of four antimicrobial families, based on a bacterial growth inhibition method [22,23]. The Four-Plate Test used included four antimicrobial families: tetracyclines, beta-lactams, aminoglycosides and macrolides, considering the formulations authorized for use in poultry in Chile [24].

An in-house laboratory validation protocol was developed according to criteria derived from the European Commission Decision 2002/ 657/CE [25]. The method was validated using spiked blank eggs, obtained from non-treated laying hens, determining the performance characteristics of the method such as specificity, detection capability, precision and ruggedness, in order to prove the fitness of the method and its applicability to the detection of antibiotic residues in eggs. The specificity of the method was in conformity with the directive with a false-positive rate of 4%.

#### 2.3.1. Preparation of Culture Media

For the analysis, cultures of *Bacillus cereus* ATCC 11778 and *Bacillus subtilis* ATCC6633 were sporulated. For these, each microorganism was seeded in a tube with antibiotic medium 1 and incubated at 30 °C for 24 h. Subsequently, 3 mL of sterile distilled water was added and transferred to a roux bottle with 250 mL of antibiotic medium 1. The culture was incubated at 30 °C for 7 days.

Then, 25 mL of sterile distilled water was added to the roux bottle and the growth was collected in sterile tubes and centrifuged at 1811 rcf for 20 min. After that, the supernatant was removed, washed with 15 mL of distilled water and centrifuged again at 1811 rcf for 20 min; this was done twice. Next, the supernatant was removed and 10 mL of 0.85% saline solution was added and heated in a water bath at 70 °C for 30 min.

*Kokuria rhizophila* ATCC9341 was activated, carrying out transfers to BHB three times, incubating it at 35 °C for 24 h each time; the third transfer was used afterward for the inoculation of the medium.

In order to evaluate the presence of residues of different families of antibiotics, four different inoculated media were designed. For this, antibiotic medium 5 and antibiotic medium 8 were prepared and autoclaved following manufacturer instructions.

For the detection of tetracycline antibiotic residues, 2 mL of a suspension of *Bacillus cereus* ATCC 11778 (concentration of 3 × 10^8^ CFU/mL) was added to 200 mL of antibiotic medium 8.

To assess the presence of aminoglycoside residues, 2 mL of a suspension of *Bacillus subtilis* ATCC6633, (concentration of 3 × 10^8^ CFU/mL), was used to inoculate 200 mL of antibiotic medium 5.

To evaluate the presence of beta-lactams residues, 3 mL of a suspension of *Kocuria rhizophila* ATCC9341 (concentration of 1.5 × 10^8^ CFU/mL) was added to 200 mL of antibiotic medium 5. Likewise, 3 mL of the same suspension was added to 200 mL of antibiotic medium 8, to evaluate the presence of macrolide residues. Subsequently, 10 mL of each inoculated medium was poured into Petri dishes. After the solidification of the agar, the metal cylinders were placed on the surface of the agar of each type of plate.

#### 2.3.2. Extraction of Antimicrobials from Samples and Inhibition Assessment

Antimicrobial residues were extracted from collected eggs in order to assess bacterial growth inhibition. The extraction procedure began by weighing in 5 g of the sample in a 50 mL polypropylene tube (protected from light). Next, 20 mL of citric-acetone buffer solvent was added. The mixture was homogenized and sonicated for 15 min twice. Then, samples were centrifuged at 1811 rcf for 15 min, and 200 uL from the resulting supernatant were poured into the metallic cylinders, placed previously on the surface of the agar plates. Subsequently, the plates were refrigerated for 30 min, and then incubated at 30 °C for 18 h. Finally, the plates were analysed by measuring the diameter of the inhibition halo using a precision Vernier caliper. If the diameter of the inhibition halo was greater than or equal to 1.2 cm, the sample was considered as positive for the presence of active antimicrobial residues.

#### 2.3.3. Positive Controls and Standards Controls

Fortified samples with oxytetracycline, erythromycin, ampicillin and amikacin at a concentration of 240, 200, 25 and 500 µg kg^−1^, respectively, were used as positive controls. These fortified samples were analysed and incubated al 30 °C for 18 h, together with the field samples.

For the control of standards, a concentration of 1000 ng mL^−1^ of oxytetracycline, erythromycin, ampicillin and amikacin were directly added to each type of inoculated medium.

## 3. Results

### 3.1. Backyard Poultry Production Characterization

A total of 83 BPS were surveyed. The median number of domestic chickens kept at BPS was 32.5 birds (IQR = 15–53). Regarding animal management (Table 1), it was possible to identify that in 77% of the BPS, women were in charge of the birds, and that 59% of the total surveyed households made sales of the products obtained from chickens, keeping part of the production for family consumption.

When evaluating the biosecurity of these systems, it was observed that 92% of the BPS raised birds together with other productive domestic animals (either pigs, cattle, or others) and only 25% of the households kept birds constantly confined, that is, in 75% of the cases, birds were free to roam in close proximity to other animals. In addition, 57% of the owners indicated that their neighbours also had birds and 23% of the owners reported that their birds had direct contact with their neighbours’ birds. Forty-nine percent of the households had a watercourse (irrigated canal, stream, river or lake edge) inside their territory and 20% of the owners declared that their birds only drank water from these environmental waters. When consulting for the management of birds, 36% of households replaced their domestic chickens with birds from abroad (that is, neighbours’, bought at fairs, among others). Additionally, 66% of the owners reported performing pharmacological treatments on their birds (whether anti-inflammatory, antibiotics and/or natural products). When asked about the management of mortalities, 53% of the BPS reported burning/burying their dead birds, while the remaining 47% indicated doing nothing, giving them to dogs or throwing the mortalities away from home. Finally, 62% of the owners indicated that they did not carry out any type of hand cleaning or disinfection prior to handling the birds, while 92% did so after handling the birds. It should be noted that 78% of households use their birds’ manure as fertilizer, and that 87% of households have an industrial poultry stock within a perimeter of 5 km.

### 3.2. Presence of Antimicrobial Residues in Backyard Poultry Eggs

From the total BPS tested, 61 were positive, and 22 BPS did not indicate the presence of any of the four tested antimicrobials, with the highest positivity for ampicillin and amikacin. It should be noted that 53 BPS were positive for more than one antimicrobial and one BPS was positive for the four antimicrobials tested. Table 2 shows the number of positive BPS for each antimicrobial family. The highest percentage of positive samples was for the families of beta-lactams, where 59.0% of the BPS were positive.

**Figure 1:**
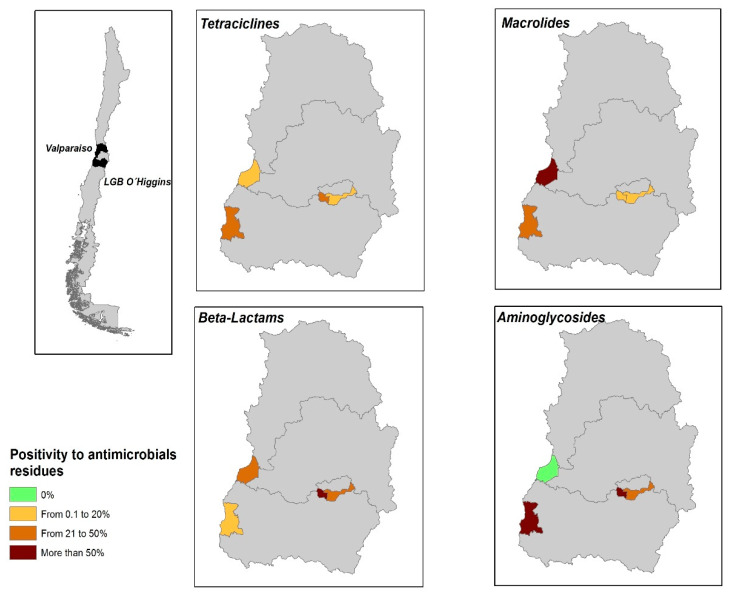
Geographical distribution of positive samples to antimicrobial belonging to families of Tetracyclines, Macrolides, Beta-Lactams and Aminoglycosides.

When considering the samples geographical distribution, we found the presence of the four tested antimicrobial families in farms located in the LGB O’Higgins region, and Tetracyclines, Macrolides and Aminoglycosides in BPS located in Valparaiso region (Figure 1).

### 3.3. Risk Factors for the Presence of Antimicrobial Residues in Backyard Poultry Eggs

The same variables collected for the BPS characterization were analyzed through univariate logistic regression to identify possible risk factors for the presence of antimicrobial residues in eggs. However, none of the variables were statistically significant (Table 3).

## 4. Discussion

The Residue Control Program for livestock products in Chile was initiated in 1987. Although the program was initiated to monitor sheep meat and hares, it has been extended to poultry, pigs, cattle, honey and dairy products [26]. However, eggs are still out of surveillance, especially when dealing with products generated in small-scale units. Nevertheless, monitoring eggs at this level is of great importance as these products are traded in local markets and intended for consumption mainly for poor people [2,4]. Complementarily, this kind of production is commonly associated with healthier products by consumers [27].

The results of this study indicate the presence of antimicrobials residues in eggs, specifically of the tetracycline, beta-lactams, aminoglycosides and macrolides families. These findings imply a risk for public health since it is highly probable that the owners of these birds have no knowledge regarding the use and impact of antimicrobial residues, or with respect to the withdrawal period [28,29], meaning that they can consume or sell these products without knowing the impact of their actions. This is especially important considering that 66% of the owners surveyed indicated giving pharmacological treatments to their chickens. Although the acquisition of antibiotics is regulated in Chile, farmers can administer remnants of antibiotics that were left in their homes after previous treatments (both for human and animal use). Thus, it is imperative to educate animal owners about the public health impact of antibiotics’ misuse.

While the route by which antimicrobials reached the eggs is not known, this could have happened by two main paths or their combination. The first would be the direct administration of antimicrobials to birds by their owners, considering that an important number of farmers indicated giving pharmacological treatments to poultry. Another hypothesis that could explain these findings would be the acquisition of antimicrobial residues by the environment [28]. This last hypothesis may be important, considering that 87% of the farms had an industrial poultry farm nearby, as well as the fact that in half of the backyards was the presence of a watercourse, which can be shared with other farms and that 78% of farmers reported the use of their own poultry manure to fertilize their cultures. Different studies have demonstrated the persistence of antimicrobial residues in soil amended with manure. Previous obtained results showed that the antimicrobial residues can persist for a prolonged period, which also depends on the concentrations and physical–chemical characteristics of the different compounds [30,31,32,33]. So, this can be a risk for the transfer of antimicrobial residues to other animals.

Our research group has identified that these animal production units represent a risk for both human and animal health, as they keep animals with low biosecurity standards, where there is also an important level of contact between different domestic species, wildlife and people [2,6,9], having antecedents of pathogens circulating in this population (Influenza A, *Salmonella* spp.) [6,7,8,9], which can directly affect the health of animals and people, as well as having repercussions on the household economy and food security [2]. There may also be a risk of direct passage of zoonotic pathogens through contact with animals, in addition to the risk of contamination during animal processing which can affect the food safety of the product [34,35].

It is important to highlight that this study was conceived as the first assessment of the presence of antimicrobial residues in eggs produced in backyard farms and that other studies are necessary to obtain, for instance, prevalence data of antimicrobial residues in eggs. Furthermore, other methods for analysis could be used, such as multiresidue chemical methods. According to this, for this first evaluation of the status of the presence of antibiotic residues in eggs, a four-plate microbiological screening method was selected. Screening methods are used to detect the presence of a substance or class of substances at the level of interest. These methods have the capability for a high sample throughput and are used to sift large numbers of samples for potential non-compliant results, and they are specifically designed to avoid false compliant results (2002/657/EC) [25]. Therefore, considering the scope of the present study, the selected methodology is suitable to provide information that allows an appropriate overview of the current status of antibiotic residues in poultry from small farms. The screening approach to detect antibiotic residues in food samples has been used for similar purposes by different authors, due to their advantages such as a short analysis time, simplicity and low cost, especially microbial screening methods, due to their high cost-effectiveness [36,37,38,39,40,41]. According to Gaudin et al. 2017, eggs are a matrix where screening methods with a wider spectrum of detection have good applicability since few methods have been developed and published for this matrix. The above-mentioned characteristics explain why this method has been selected by different authors to carry out similar studies in eggs in developing countries such as Nigeria, Bangladesh and Pakistan [42,43,44].

Despite positive characteristics that allow the use of this methodology to study the presence of antibiotic residues in food matrices, particularly in eggs, microbiological methods do not allow to identify the antibiotic residue present in the sample, identifying the presence of an antibiotic family. Microbiological methods are qualitative methods giving a response as negative, positive or doubtful [36,45]. Therefore, their applicability depends on the scope of the study and its final objective. For this first assessment of antibiotic residues in backyard poultry eggs, the selected method is fit for purpose, providing valuable information on the presence or absence of four different antibiotic classes in these products, becoming a baseline for the development of further research.

The obtained results expose the need of thorough study of the presence of antibiotics in eggs from backyard production. For a second stage, the microbiological method can be used as a screening method for the presence of antibiotics in chicken eggs, and afterwards positive samples can be confirmed and quantified using Liquid Chromatography. This approach has also been used by other authors for the detection and quantification of different antibiotics in eggs and other food products [40,41,46]. Furthermore, in the study performed by Shahbazi et al. (2015), authors compare the results obtained with the four-plate microbiological method and the Liquid Chromatography method, obtaining a correlation of 0.940, showing that microbiological inhibition tests had a high sensitivity for antibiotic detection; however, it cannot quantify the residues. Thereby, identification and quantification of the antibiotics should be considered in a follow-up study [40].

Finally, the results of this study identified antibiotic residues in most of the farms studied, including all the classes of antibiotics used. In this sense, it can be speculated that the levels of use of drugs is higher than that reported by producers in central Chile, or that there may be some effect of environmental contamination given, for example, by the use of antibiotics in intensive animal production systems in the proximity of BPS, which may be contaminating water, soil or inputs used to feed birds in BPS [29,47]. It would be a great contribution to the scientific community, so future research aims to raise more information regarding environmental pollution given by the use of antibiotics, as well as improving efforts in collecting more information on the obtention and use of antibiotics by these producers.

## 5. Conclusions

Eggs produced in backyard farms in central Chile are carrying antimicrobial residues of the four tested antimicrobial families (Tetracyclines, Macrolides, Beta-lactams and Aminoglycosides). Its role in antimicrobial resistance spread and the specific risk for food safety must be addressed for eggs produced in backyard farms, focusing on the route by which antimicrobials reached the eggs. This research in backyard poultry eggs provides baseline information for this non-addressed food safety issue, and demonstrates that further studies are needed. The next steps should include the confirmation and quantification of the pharmacologically active substances in eggs, in order to identify the antibiotics that are being used and assess the risk that this may represent to the consumers.

## Figures and Tables

**Table 1:** Variables considered for the characterization of poultry handling and biosecurity conditions in BPS that keep poultry in central Chile.

Farm Management	Definition and Classification	%
Other domestic animals	Presence of other domestic animals in the BPS	92.0
	Absence of other domestic animals in the BPS	8.0
Declared use of poultry	Sale and household consumption	59.0
	Household consumption	41.0
Poultry management	Man is in charge of poultry management	23.0
	Woman is in charge of poultry management	77.0
Feeding	Scavenging, household scraps and supplemented with poultry feed and grains	53.0
	Scavenging and household scraps	47.0
Poultry housing	Poultry are permanently confined during the day	25.0
	Poultry are partially confined during the day	75.0
Guano handling	Use guano as fertilizer	78.0
	Does not use guano as fertilizer	22.0
Poultry in neighbour’s	Neighbours have poultry	57.0
	Neighbours do not have poultry	43.0
Poultry/neighbour’s poultry	Poultry do contact neighbours’ poultry	23.0
	Poultry do not contact neighbour’s poultry	77.0
Replacements	Replace poultry from their own offspring	64.0
	Replace poultry from places outside their BPS	36.0
Water	Poultry drink potable water	81.0
	Poultry get water from environmental sources	19.0
Mortality handling	Bury or burn dead poultry	53.0
	Do not bury or burn dead poultry	47.0
Pharmacotherapy	Give pharmacological treatment to poultry	66.0
	Do not give pharmacological treatment to poultry	34.0
Disinfection prior handling	Wash hands before handling poultry	38.0
	Do not wash hands before handling poultry	62.0
Disinfection post handling	Wash hands after handling poultry	92.0
	Do not wash after before handling poultry	8.0
Water body	Existence of a water body in a radius of 5 km around the BPS	49.0
	No existence of a water body in a radius of 5 km around the BPS	51.0
Commercial farm nearby	Existence of a commercial farm within a radius of 5 km around the BPS	87.0
	Nonexistence of a commercial farm within a radius of 5 km around the BPS	13.0

**Table 2:** Positivity of backyard productive systems (BPS) to antimicrobials residues in central Chile, 2019.

Antimicrobial	Positive BPS (Nº/%)
Tetracyclines	17/20.5
Macrolides	11/13.3
Beta-Lactams	49/59.0
Aminoglycosides	47/56.6

**Table 3:** Results of logistic regression to identify possible risk factors for the presence of antimicrobial residues in eggs in BPS from central Chile.

Variable	O.R.	95% CI	*p*-Value
Constant	12.75	0.2–807.42	0.2292
Presence of other domestic animals	0.49	0.02–9.84	0.6403
Poultry management in charge of woman	0.4	0.06–2.64	0.3394
Constant poultry housing	10.64	0.9–125.52	0.0604
Do not use guano as fertilizer	0.87	0.13–5.85	0.8888
Poultry contact neighbour’s poultry	4.39	0.72–26.67	0.1085
Replacements from own offspring	1.76	0.41–7.65	0.4457
Poultry drink potable water	0.29	0.04–1.9	0.1957
Mortality is buried/burnt	1.99	0.44–8.98	0.3713
Poultry receives pharmacotherapy	0.64	0.13–3.04	0.5735
Presence of a water body 5 km nearby	0.41	0.1–1.71	0.2197
Number of birds	1.00	0.99–1.01	0.711
